# Association between Physical Activity, Diet Quality and Leisure Activities of Young Poles

**DOI:** 10.3390/nu15245121

**Published:** 2023-12-16

**Authors:** Marta Sajdakowska, Krystyna Gutkowska, Małgorzata Kosicka-Gębska, Jerzy Gębski, Andrzej Gantner

**Affiliations:** 1 Department of Food Market and Consumer Research, Institute of Human Nutrition Sciences, Warsaw University of Life Sciences (SGGW-WULS), 159C Nowoursynowska Street, 02-787 Warsaw, Poland; krystyna_gutkowska@sggw.edu.pl (K.G.); malgorzata_kosicka_gebska@sggw.edu.pl (M.K.-G.); jerzy_gebski@sggw.edu.pl (J.G.); 2 Polish Federation of Food Industry Union of Employers, 19B Cybernetyki Street, 02-677 Warsaw, Poland; a.ganter@pfpz.pl

**Keywords:** physical activity, adolescents, diet quality, leisure activities

## Abstract

The aim of this study was to determine the association between eating behaviours, sedentary behaviours and physical activity based on a self-reported survey conducted on a sample of the Polish population of adolescents aged 13–16. The field survey was conducted on a nationwide group of 6818 respondents. The object of the statistical analysis was to develop a model regarding the influence of selected socio-demographic characteristics on engaging in physical activity and selected dietary behaviours. Due to the dichotomous nature of the dependent variable, logistic regression models were used in the model. It was found that the higher the level of physical activity, the more well-balanced the diet, including higher levels of fruit consumption, water consumption and protein-containing products, as well as the relatively less frequent occurrence of sedentary behaviour as a form of leisure activity. However, there is a constant need to develop, in cooperation with scientific and research institutions and educational establishments, mechanisms for influencing a change in the behaviour of young people towards a more pro-healthy lifestyle so that the effects of these educational activities are not only reflected in an increase in the level of knowledge in this area but also contribute to real changes in dietary behaviour.

## 1. Introduction

The findings of the research on lifestyle demonstrate the relative coherence of the behaviours constituting it, which is due, among other things, to the fact that behaviours are the result of recognised values, between which there is also a relative consistency [1,2]. Lifestyle is defined by the World Health Organisation (WHO) as the choice of behavioural patterns among those available to a person that are determined by socio-economic factors and the type of motivation that influences the choice of some behaviours over others [3]. Assuming the relative consistency of the types of motivation that may determine or foster health-promoting behaviours as a result of the recognition of health as an important value in the axiological system of each individual [4], it can be indicated that, among others, proper nutrition, physical activity, coping with stress, attention to personal hygiene, proper interpersonal relationships and regular preventive examinations or attention to the quality of sleep will be components of a lifestyle that is perceived in the literature as healthy [5,6,7].

Physical activity is a variety of activities involving movement and skeletal muscle activation, resulting in an energy expenditure that is higher than at rest [8]. It is an essential part of everyone’s life and is particularly important for children and adolescents. The World Health Organisation recommends at least 1 h of moderate to vigorous exercise for children and adolescents aged 5–17. In addition, at least three times a week, a child should perform intensive exercise for 30 min, during which the muscles responsible for posture are strengthened, as well as perform exercises to improve flexibility, speed and muscle strength [9]. Physical activity associated with movement is a fundamental factor in the regularity of developmental processes. It reduces the risk of many diseases in adulthood. Scientific research has shown that it ensures the correct development of the musculoskeletal system and stimulates the development of the cardiovascular and respiratory systems as well as the development of the nervous system. It is also a prerequisite for maintaining a healthy body weight. Exercise is also a hardening factor for children as it stimulates their immunity and contributes to the prevention of various childhood diseases. It also influences a young person’s mental condition. Movement and physical exercise have a positive effect on mood, make it easier to cope with stress and support the treatment of depression [9,10,11,12].

Despite the numerous benefits of physical activity outlined above, there has been a decline in physical activity rates among children and young people in Poland in recent years [13]. This status quo has been confirmed by the findings of the HBSC (Health Behaviour in School-aged Children) study of the health behaviour of schoolchildren, which has been conducted in Poland every 4 years since 1990 [14]. Data published in the 2017–2018 report by the Public Health Committee of the Polish Academy of Sciences and the National Institute of Public Health—National Institute of Hygiene—Health Behaviour in School-aged Children also leave no doubt about the decrease in physical activity among young people. Only 15.6% of students aged 11–18 met the WHO criteria related to daily exercise for a minimum of 60 min. Recommendations to exercise intensively four times a week were met by 31.0% of students [15]. Insufficient levels of physical activity among children and adolescents have also been observed in international studies [16,17,18].

There are many reasons for low physical activity among children and adolescents. One of them may be incorrect physical activity habits acquired in childhood, with parents, teachers and peers as role models [19,20]. Another determinant of low physical activity is the widespread availability of various forms of transport (e.g., cars, lifts, escalators) replacing the need for moving around personally. In families with fewer economic opportunities, it is more difficult to meet the need to participate in various forms of physical activity [21]. Another reason for this situation may also be the increasing reluctance of students to participate in physical education classes as they become older [14]. The study ‘Social diagnosis of students 2018’ [22] found that 14% of students only undertook physical activity during PE lessons. 

Low levels of physical activity in children and adolescents, the prevalence of sedentary lifestyles and poor eating habits are the causes of increasing health problems, including an epidemic of obesity, type 2 diabetes in increasingly younger people and cardiovascular disease [23]. A number of studies indicate that adolescents and children are obese as a result of sedentary behaviours that dominate during the 24 h period [24]. Watching TV, sitting in front of a computer screen or using the possibilities offered by the phone replaces taking up physical activity by children and adolescents in their free time [25]. The WHO recommends that children and adolescents limit time spent sitting, especially in front of screens (phones, tablets, computers) [9]. It was found that through setting a personal example, consistent decision making in the area of rational leisure time management, parents can effectively influence a reduction in sedentary behaviour in younger children [26]. Moreover, adolescents in puberty often engage in health and physical activity risk behaviours. These include, for example: drinking alcoholic beverages, smoking, using prohibited substances, risky sexual behaviour, sedentary lifestyles and poor diet [20]. The dietary choices that children and young people make are influenced by a number of factors. These range from individual factors, e.g., one’s own dietary attitudes and preferences, to norms from the social environment, especially family and peers, as well as the influence of the wider environmental context: food marketing, dietary policies, the regulation of school feeding rules or the availability of food products [27].

The formation of physical activity habits is significantly influenced by government policy. According to Article 68 section 5 of the Constitution of the Republic of Poland of 2 April 1997, public authorities shall support the development of physical culture, especially among children and young people [28]. In 2016–2019, the Ministry of Sport and Tourism implemented a number of programmes in Poland concerning the development of physical activity among children and young people as part of the Sport Development Programme 2020. However, the results of verifying the effectiveness of these programmes by the Supreme Chamber of Control (NIK) showed that they did not increase physical activity among children and young people in Poland [29].

It therefore seems important to introduce effective measures, including educating children and adolescents about improving physical activity and providing them with easy access to sports facilities. Indeed, the study found that students who undertook daily physical activity rated their physical fitness and their own health very well at the same time. Young people undertook physical activity for reasons of perceived enjoyment, good health, attractive appearance, body care and stress relief. Higher frequencies of physical activity were demonstrated by boys, students achieving higher grades at school and children of parents with secondary and higher levels of education. Motivations related to sport participation were significantly differentiated by gender. Girls were more likely than boys to participate in sport in order to look good, have a nice figure, relieve stress or be in good shape. Boys, on the other hand, exercised more frequently for the pleasure they experienced and for their health [30]. Furthermore, young people involved in sport are a group with specific nutritional needs. High physical activity combined with intensive developmental processes increase the demand for energy and macro- and microelements. In the nutrition of adolescents involved in physical activity, a rational, well-balanced diet is of particular importance. On the basis of the analysis of research findings, it may be concluded that the level of knowledge of young people involved in physical activity concerning the principles of proper nutrition is insufficient [31], although higher than that of the physically inactive ones [1]. Children and adolescents in many countries consume too few fruits and vegetables, over-consume products high in sugar, including sweets and sweetened beverages [32], and the cereal products chosen are not whole grain [33]. It has also been noted that, among young people involved in a variety of sports, protein-containing products (e.g., meat, eggs, legumes) are more likely to be introduced into the daily diet [34].

Although the available evidence for the existence of associations between dietary habits and physical activity is quite strong for children and adolescents [35,36,37], to the knowledge of the authors of this study, there is still a need to re-analyse and update research in this area, as many factors may be relevant here. Therefore, the aim of this study was to determine the association of eating behaviours, sedentary behaviours and physical activity based on a self-reported survey conducted on a sample of the Polish population of adolescents.

## 2. Materials and Methods

### 2.1. Data Collection Process

The research presented in this article is part of a larger research project, the details of which in terms of sampling for the study and analysis of the findings for the overall research sample were discussed in earlier papers [1,38,39]. The primary study included a quantitative survey on “Evaluation of the effectiveness of the National Educational Program “Keep Fit!” in terms of shaping the eating and health habits of its participants”. The aim of the study was to examine the daily behaviour of adolescents aged approximately 13–16 years connected with nutrition, hydration and physical activity. The field survey was conducted in 2016 on a nationwide group of 6818 students. The quota selection method involves selecting a group of respondents in a proportion corresponding to the structure of the general population of students in lower secondary schools in Poland. 

The selection of typical units was performed, i.e., segmentation of respondents from a particular community as per:

- Students from schools following the National Educational Programme “Keep Fit!” and those directly engaged in the launching of the scheme (50.38%);
- Students from schools following the National Educational Programme “Keep Fit!” but not engaged in the scheme (34.44%);
- Students from schools not following the Programme and not engaged in the scheme (15.18%).

### 2.2. Description of Questionnaire and Data Analysis

An online survey questionnaire made up of several parts was carried out to obtain empirical material. A school representative completed the first section of the questionnaire with details such as: the institution’s data, the size of the town in which the school is based and whether the school participates in the National Educational Programme “Keep Fit!”. In the next step, students provided their responses. In this way, the collected data were credible regarding students’ affiliation to a given educational institution. 

The questions generally pertained to several thematic blocks, including: evaluation of diet; evaluation of physical activity; evaluation of the extent of consumer awareness, including, among others, evaluation of the use of selected information on food packaging. The second part of the survey questionnaire comprised a set of questions that enabled one to characterise the respondents in terms of the socio-demographic characteristics. 

In order to achieve the assumed research goal, the article presents data relating to selected aspects of the study referring to the diet, sedentary behaviour and physical activity, i.e., questions enabling the assessment of:

- The consumption of certain types of food (fruit; vegetables; cereals, e.g., bread, pasta, groats, rice; milk and dairy products; other products containing protein, e.g., meat, meats, legumes, eggs);
- The frequency of water consumed during the day;
- The amount of time devoted to physical activity at and outside school;
- The amount of time spent using devices with TV screen or electronic devices (e.g., a computer, a tablet, a smartphone).

The survey questionnaire was effectively completed by 6504 people aged 13–16. Socio-demographic characteristics of the sample using frequency analysis were used for preliminary analysis of the results. The object of the actual statistical analysis was to develop a model regarding the influence of selected socio-demographic characteristics and engaging in physical activity and selected dietary behaviours. 

In the model, the dependent variable was physical activity. Due to the dichotomous (binary) dependent variable, logistic regression models were used in the model. 

The levels of the dependent variable were as follows:

- (1) Not undertaking physical activity at all, (2) undertaking physical activity for a total of 60 min per day 1–2 days per week;
- (1) Undertaking physical activity for a total of 60 min a day on 3–4 days a week, (2) 5–6 days a week and (3) daily.

The study made a prediction of undertaking physical activity. Among the independent variables in the model (explanatory variables; statistically significant variables included in the model), there were:

- Eating meals during so-called school days;
- Consumption of protein/protein products;
- Consumption of fruit;
- Frequency of drinking water;
- Frequency of playing computer/console games;
- Frequency of PC use during school days;
- Frequency of PC use during weekends.

All variables were statistically significant in the model at *p* < 0.05. The validity of model was confirmed by model fit statistics and the Hosmer and Lemeshow test (*p* = 0.7988). The statistical analysis was performed using the SAS 9.4 statistical package (SAS Institute, Cary, NC, USA).

## 3. Results

### 3.1. Description of the Sample

The majority of young respondents participating in the study declared that they were physically active. Among those declaring daily physical activity, over 70% of respondents indicated that they eat at least four meals a day. Taking into account the class attended by children and adolescents, it occurred that, each time, a larger group of respondents, regardless of the class they attended, declared eating at least four meals a day. Taking into account the class attended by the respondents indicates that the majority of students in the study were second and third grade students of junior high school. Among the respondents participating in the study, a slightly larger percentage participated in The National Educational Program “Keep Fit!” (%) compared to people declaring that they did not participate in it (%) (Table 1).

### 3.2. Physical Activity among Adolescents

For those who never eat breakfast at home, the risk of being inactive (one to two times a week at most) increases by 36% relative to those who eat breakfast daily (OR: 1.36; 95% CI: 1.15–1.61). And, for those who eat breakfast occasionally, the risk of being inactive increases by 20% relative to those who eat breakfast daily (OR: 1.20; 95% CI: 1.04–1.39) (obviously keeping the other model parameters constant).

When protein-containing products are consumed more frequently, the risk of being inactive decreases, with those eating protein products two times a day having a 35% reduced risk compared to those eating them once a day (OR: 0.65; 95% CI: 0.56–0.75). For those eating three protein meals per day, the risk decreases by 43% (OR: 0.57; 95% CI: 0.47–0.69), for those eating four portions of protein products per day, by 46% (OR: 0.54; 95% CI: 0.41–0.72) and for those eating five portions with a high protein content, by 37% (OR: 0.63; 95% CI: 0.49–0.80) relative to the reference level (one portion per day). With a higher frequency of fruit consumption, the chance of being inactive decreased. For those eating fruit two times a day, the chance of being inactive decreased by 7% compared to those eating one portion a day (OR: 0.93; 95% CI: 0.79–1.09), but this level of variable was not statistically significant. For those eating fruit three times a day, the risk of being inactive decreased by 11% (OR: 0.89; 95% CI: 0.75–0.99), for those eating four portions of fruit a day, by 32% (OR: 0.68; 95% CI: 0.54–0.87) and for those eating fruit five times a day, by 17% (OR: 0.83; 95% CI: 0.66–0.94).

A lower frequency of water drinking influenced the increased risk of being inactive. Those who did not drink water at all had a 228% higher risk of being inactive compared with the reference level (drinking more than five times a day) (OR: 3.28; 95% CI: 2.54–4.23). Those drinking water once a day had a 171% higher risk of being inactive compared to baseline (OR: 2.71; 95% CI: 2.17–3.39). Those drinking water two times a day had an 89% higher risk compared to the reference level (OR: 1.89; 95% CI: 1.54–2.31), those drinking water three times a day had a 63% higher risk (OR: 1.63; 95% CI: 1.35–1.96) and those drinking water four times a day had a 19% higher risk compared to the reference level (OR: 1.19; 95% CI: 1.07–1.45). Those drinking water five times a day had a 17% higher risk of being inactive than those drinking water more than five times a day, but this level of variable was not statistically significant in the model.

Participation in games (so-called “computer” games, etc.) on school days only for those playing for 5 or more hours per day resulted in a 60% increased risk of being inactive outside of school (OR: 1.60; 95% CI: 1.22–2.10). The remaining levels of this variable were not statistically significant within the model.

Longer PC use on school days was correlated with a reduced risk of being inactive. Those using the PC 0.5–2 h per day had a 24% lower risk of being inactive than those not using the PC at all (OR: 0.76; 95% CI: 0.51–0.98). Those using PC on school days 3–4 h a day had a 42% lower risk of being inactive (OR: 0.58; 95% CI: 0.39–0.87) and those using 5 or more hours a day had a 39% lower risk of being inactive (OR: 0.61; 95% CI: 0.40–0.92) compared to the reference level (not at all/not using at all).

For PC use at weekends, this variable is statistically significant in the model developed, but no level showed statistical significance (Table 2).

## 4. Discussion

### 4.1. The Importance of Diet Quality and Leisure Time in Increasing the Level of Physical Activity

Analysis of the results of our own research has shown that eating breakfast at home rarely and not eating breakfast at all increases the risk of low physical activity in children and adolescents. The authors’ research indicates that about one third of Polish pupils aged 11–13 years do not eat breakfast every day [40]. Larson et al. (2013) highlights that eating meals together with the family addresses a number of nutritionally beneficial choices for children and adolescents (diet quality), e.g., a higher consumption of fruit and whole grain products, as well as resulting in a lower incidence of obesity/overweight [41]. It is also generally noted in the literature that although breakfast consumers had significantly higher diet quality scores and better nutrient intakes than breakfast skippers, on average, both groups had poor diet quality. Consequently, it is unlikely that simply advising teens to consume breakfast will result in a meaningful change in diet quality, and more effort should be placed on promoting nutritious breakfasts. It is therefore important to encourage young people to eat a well-balanced (nutritious) breakfast rather than just pointing out that breakfast is worth eating [42]. In addition, the findings of other studies contribute to the evidence suggesting that eating breakfast more frequently is associated with higher levels of physical activity [43].

Our own research has also shown that, with an increase in the amount of meals containing protein valuable to the young, growing organism (i.e., meat, cold cuts, legumes, eggs), the risk of low physical activity among children and adolescents decreases. Studies by other authors focusing on the dietary behaviours of physically active individuals note the consistency of sport-related behaviours with broader positive dietary behaviours, i.e., for example, the way in which purchasing decisions are made and the awareness that a well-planned diet is a support for any appropriately chosen training strategy [44]. Bączkowicz et al. (2007) [45] state that physically active consumers have much knowledge about what they buy and what food products they consume; consumers from this group also know what nutritional properties a given product has and how it affects their body. As Gabryś (2018) [46] points out, based on the research conducted, people who have been exercising for several months, a dozen or so months or several years have changed their habits, customs and routine, while also changing their lifestyle. On the other hand, the lack of physical activity is related to cravings for sweets, using food as a reward and for pleasure, that may lead to meal skipping and a higher intake of sweets instead of other products [47]. In addition, studies by other authors note a correlation between a healthy diet and undertaking physical activity by adolescents [1,48,49].

Our study also found that as the frequency of fruit consumption increased, the risk of being inactive decreased, which was also confirmed by the results of other studies [1,50,51,52,53]. In studies conducted among Polish adolescents engaged in physical activity, it was noted that recommendations for the daily consumption of several portions of fruit were fulfilled by more girls/teenagers (more than half of the sample) than boys/teenagers (about one-fifth of the sample). Responses concerning vegetable consumption accounted for an even smaller share. A low consumption of vegetables and fruit significantly reduces the supply of antioxidants, which are of key importance in the nutrition of athletes due to the phenomenon of oxidative stress that develops under conditions of intense exercise [31].

With regard to the level of water consumption, our research showed that as the frequency of water consumed by children and young people increased, the risk of being inactive decreased. Water is directly consumed by humans as an ingredient in beverages and food. The overall water content of the human body is between 50 and 75% of body weight. Water intake is important in preventing dehydration and the onset of many diseases [54]. Among other things, it is recommended for the prevention of obesity [55]. Thus, on the 2005–2006 National Health and Nutrition Examination Survey (NHANES), the average total water intake among US youth (aged 12 to 19 years) was 2.4 L/day (1.6 L/day from food and beverages other than drinking water and 0.8 L/day in plain water) [56]. The water requirements of physically active people are higher compared to those who do not engage in physical activity [54]. However, there is relatively little research indicating an association between water drinking and physical activity among children and adolescents [57,58]. It was observed that students distinguished by their low water intake were less likely to consume 100% juices and milk, and more likely to consume fizzy drinks and sweetened fruit drinks. It was concluded that a low water intake may be associated with a higher consumption of less health-promoting products [54]. In our study, as indicated earlier, as in the other study [56], it was proven that as the frequency of water consumed by children and adolescents increased, there was a decrease in the risk of being inactive. Passive leisure activities of watching television contribute to a low consumption of pure water and high consumption of sweetened drinks [54]. Moreover, sugar drinks are the largest source of added sugar. A high consumption of sugar drinks has been associated with obesity among youth [59].

In light of the results obtained in our own study, it can be concluded that the more time adolescents spent playing PC games on school days (those playing 5 or more hours a day), the more their physical activity outside school decreased. At the same time, however, our study showed that, overall, a longer time spent on PC use on school days was associated with a reduced risk of being inactive, which may most likely be related to better planning and use of the time available to adolescents during the so-called working week. Goldfield et al. [60] indicates that time spent in front of a screen (screen time duration) and time spent playing various types of electronic games, as well as recreational computer use (i.e., computer use for pleasure), results in a lower, in relation to health, quality of life among young people. Moreover, results of other studies suggest that parents’ encouragement and support can increase their children’s physical activity and reducing the parents’ own screen time can lead to a decreased child screen time. Improving parenting practices, parental self-efficacy and parenting style may also be promising approaches to increasing the physical time and decreasing the screen time of young children [61]. In addition, studies by other authors indicate that sedentary behaviour, low physical activity and unhealthy behaviour in relation to food and nutrition (dietary patterns) are risk factors for many chronic diseases, e.g., obesity [62]. In order to modify the leisure behaviour of children and adolescents, the need to reduce the amount of time spent using various electronic tools and time spent in front of the television is indicated [63], especially since watching television was associated with more favourable attitudes towards unhealthy foods [64]. Consideration of gender indicates that girls are more likely to care about their own health and physical appearance compared to boys [30]. However, it has also been noted that girls, especially those living in urban areas, should be targeted by public health improvement initiatives to prevent obesity and increase overall youth well-being [65].

### 4.2. Strengths and Limitations 

The strength of the study is the large size of the research sample. However, similar to all studies, the present study also has some limitations. The first concerns self-reported information obtained from the questionnaire that may be inaccurate due to the unnatural situation created by the questionnaire itself. Moreover, the aspect worth paying more attention to in the future when designing further surveys among children and young people is young people’s use of various modern electronic tools (e.g., smartphone, tablet, computer/PC), e.g., for both educational purposes and the development of interests among children and young people, as well as for leisure activities in the broader sense.

## 5. Conclusions

In the context of the research results presented, it can be concluded that the relationship assumed in the title of the article between physical activity, diet quality and leisure time activities was confirmed, proving the interdependence of the occurrence of these behaviours as connoting a pro-healthy lifestyle. It was found that, in the category of children and adolescents aged 13–16 years, the higher the level of physical activity, the more well-balanced the diet, including higher levels of fruit consumption, water consumption and the intake of protein-containing products, as well as the relatively less frequent occurrence of sedentary behaviour as a form of leisure activity.

The low level of physical activity diagnosed by numerous researchers and, at the same time, the markedly higher level of physical activity among young people participating in various nutrition education programmes prove the validity of carrying out consistent actions of this kind. There is also a need to develop, in cooperation with scientific and research institutions and educational establishments, including teachers, mechanisms for genuinely influencing a change in the behaviour of children and young people towards a more pro-healthy lifestyle so that the effects of these educational activities are not only reflected in an increase in the level of knowledge in this area but also contribute to real changes in dietary behaviour.

## Figures and Tables

**Table 1:** Sample profile (%, N).

		Number of Meals Consumed per Day			
Variable	Total	1, 2, 3	4, 5 and More	Total	*p*-Value
physical activity during the last week	not a single day	99	146	245	<0.0001
		40.41	59.59		
	several days a week	1226	3196	4422	
		27.73	72.27		
	daily	487	1350	1837	
		26.51	73.49		
gender	male	804	1999	2803	0.1972
		28.68	71.32		
	female	1008	2693	3701	
		27.24	72.76		
class/age	1st grade of junior high school/13–14 years old	537	1214	1751	0.0069
		30.67	69.33		
	2nd grade of junior high school/14–15 years old	648	1810	2458	
		26.36	73.64		
	3rd grade of junior high school/15–16 years old	627	1668	2295	
		27.32	72.68		
participation in the programme	yes	852	2623	3475	<0.0001
		24.52	75.48		
	no	960	2069	3029	
		31.69	68.31		

Test of independence *χ*^2^ *p* < 0.05; N = 6504.

**Table 2:** Statistically significant variables and their estimation properties used to build the logistic regression model.

Variable	Variable Level	Estimate	Point Estimate	95% Wald Confidence Limits		*p*-Value
Intercept		−0.867				0.0006
Breakfast on school days	I never eat breakfast	0.306	1.36	1.15	1.61	0.0003
	I sometimes eat breakfast	0.184	1.20	1.04	1.39	0.0119
	I eat breakfast every day (ref.)	0	1			
Product consumption—protein-containing products	two portions per day	−0.439	0.65	0.56	0.75	<0.0001
	three portions per day	−0.561	0.57	0.47	0.69	<0.0001
	four portions per day	−0.611	0.54	0.41	0.72	<0.0001
	five portions per day	−0.466	0.63	0.49	0.80	0.0002
	one portion per day (ref.)	0	1			
Product consumption—fruit	two portions per day	−0.076	0.93	0.79	1.09	0.3543
	three portions per day	−0.115	0.89	0.75	0.99	0.0418
	four portions per day	−0.383	0.68	0.54	0.87	0.0016
	five portions per day	−0.188	0.83	0.66	0.94	0.0313
	one portion per day (ref.)	0	1			
Drinking water	I don’t drink water at all, I don’t like it	1.188	3.28	2.54	4.23	<0.0001
	once a day	0.996	2.71	2.17	3.39	<0.0001
	twice a day	0.634	1.89	1.54	2.31	<0.0001
	three times a day	0.488	1.63	1.35	1.96	<0.0001
	four times a day	0.170	1.19	1.07	1.45	0.0459
	five times a day	0.154	1.17	0.93	1.47	0.1896
	more than five times a day (ref.)	0	1			
How much he/she plays games—during school days	0.5–2 h per day	0.006	1.01	0.88	1.15	0.9317
	3–4 h per day	0.046	1.05	0.84	1.30	0.6795
	5 and more hours per day	0.471	1.60	1.22	2.10	0.0007
	at all (ref.)	0	1			
How much he/she uses a PC—on school days	0.5–2 h per day	−0.279	0.76	0.51	0.98	0.0478
	3–4 h per day	−0.546	0.58	0.39	0.87	0.0083
	5 and more hours per day	−0.501	0.61	0.40	0.92	0.0182
	at all (ref.)	0	1			
How much he/she uses a PC—at weekends	0.5–2 h per day	−0.275	0.76	0.46	1.25	0.2809
	3–4 h per day	−0.189	0.83	0.5	1.37	0.4621
	5 and more hours per day	0.022	1.02	0.61	1.7	0.9336
	at all (ref.)	0	1			

Point estimate—OR (eβ); (95% Cl)—95% Wald confidence limits; ref.—reference group; N = 6504.

## Data Availability

Data are contained within the article.
